# Unusual Case of Coexisting Renal Malignancies: Mucinous Tubular and Spindle Cell Carcinoma Kidney With Sarcomatoid Dedifferentiation

**DOI:** 10.15586/jkcvhl.2016.51

**Published:** 2016-05-31

**Authors:** Kafil Akhtar, Pragati Agnihotri, Kiran Alam, Kashif Raza

**Affiliations:** 1 Department of Pathology, and 2 Department of Radiotherapy, Jawaharlal Nehru Medical College, Aligarh Muslim University, Aligarh, UP, India

**Keywords:** immunohistochemistry, kidney, mucinous, sarcomatoid dedifferentiation, spindle cell carcinoma

## Abstract

Mucinous tubular and spindle cell carcinoma (MTSCC) is a recent entity introduced in the World Health Organization 2004 Classification. It is a tumour of low malignant potential. MTSCC is a subtype of renal cell carcinoma (RCC), which is characterized by a polymorphous histology, wherein the spindled epithelial cell is an inherent carcinomatous component. We report the case of a 57-year-old man presenting with loin pain and dragging sensation. Imaging revealed a large mass arising from the left kidney. Radical nephrectomy was performed, and histopathology revealed spindle cell elements of MTSCC with low-grade cytology, which occasionally blended with tubular structures in variable mucinous stroma admixed with spindle sarcomatoid cells with marked nuclear pleomorphism, associated with significant necrosis and mitoses of up to 5/10 high-power field. A final diagnosis of MTSCC along with high-grade areas consistent with sarcomatoid dedifferentiation was made. Sarcomatoid dedifferentiation has been well documented in various subtypes of RCC, and its presence signifies a worse prognosis in RCC.

## Introduction

Mucinous tubular and spindle cell carcinoma (MTSCC) is a low-grade renal epithelial neoplasm accepted as an individual entity in the World Health Organization (WHO) 2004 Classification (1). MTSCC has a less malignant potential than other subtypes of RCC, and so it is of prognostic and therapeutic importance to distinguish it from the others so as to avoid unnecessary adjuvant immunotherapy with interferon-α and interleukin-2 (1, 2).

Histologically, the tumour consists of anastomosing tubules of cuboidal and spindle cells with low-grade nuclei (2, 3). Staining with alcian blue reveals lakes of mucin. Immunohistochemical studies show that MTSCC is positive for the markers of epithelial cells and distal nephron and negative for proximal nephron, i.e., epithelial membrane antigen^+^ (EMA^+^), antibody elexa (AE1/AE3^+^), cytokeratin 7^+^ (CK7^+^), CK19^+^, E-cadherin^+^, α-methylacyl-CoA racemase^+^ (AMACR^+^) and cluster designation 10^−^ (CD10^−^) (4). Sarcomatoid dedifferentiation (SD) is seen in many renal cell carcinomas (RCCs), which is usually associated with a poorer outcome. Tumours containing SD have a decreased 5-year survival rate from 79% to 22% in stage-matched patient cohorts; tumours containing >50% SD have an even worse prognosis (4). The areas of SD are markedly different from the spindle cell component of the MTSCC portion of the tumour, both histologically and immunohistochemically, confirming that the spindle cell component seen is part of the MTSCC and not the SD portion of the tumour (5). High-grade cytology, expansile growth, extensive necrosis, high mitotic activity, high proliferation fraction and loss of expression of α-methylacyl-CoA racemase are some helpful features in distinguishing spindle cells of the sarcomatoid component from that of the native tumour. We report a rare case presenting with MTSCC with SD, the combination of which has a worse prognosis.

## Case summary

Approval from institutional Ethics Committee was obtained. A 57-year-old male patient presented with pain and dragging sensation in the left loin along with weight loss. Computed tomography examination of abdomen showed a large cystic mass of size 17 × 11 × 24.6 cm arising from the middle and upper poles of the left kidney. No abdominal lymphadenopathy was observed. Ultrasound of the scrotum and colonoscopy findings were unremarkable. Beta-human chorionic gonadotropin (HCG), serum lactate dehydrogenase (LDH), carcinoembryonic antigen (CEA) and alpha fetoprotein (AFP) values were within normal limits. Left radical nephrectomy was performed.

**Figure 1.**
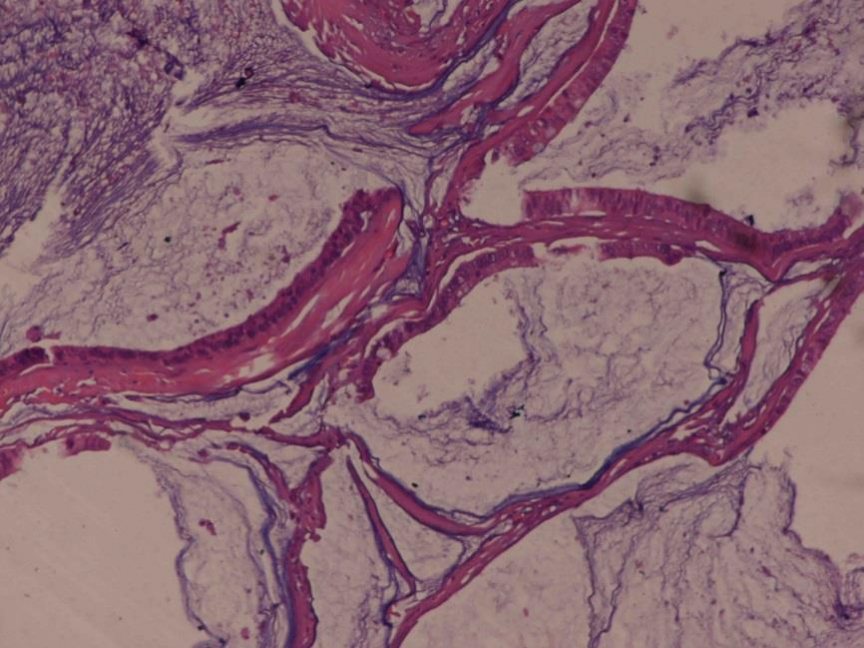
Microscopically, the tumour consisted of elongated, anastomosing tubules separated by lakes of mucin from a prominent spindle cell area. The tubules were composed of low cuboidal cells with an amphophilic to eosinophilic cytoplasm with low-grade nuclei. Foci of sarcomatoid differentiation were seen with pleomorphic high-grade nuclei without any evidence of vascular invasion. Haematoxylin and eosin, 10×.

**Figure 2.**
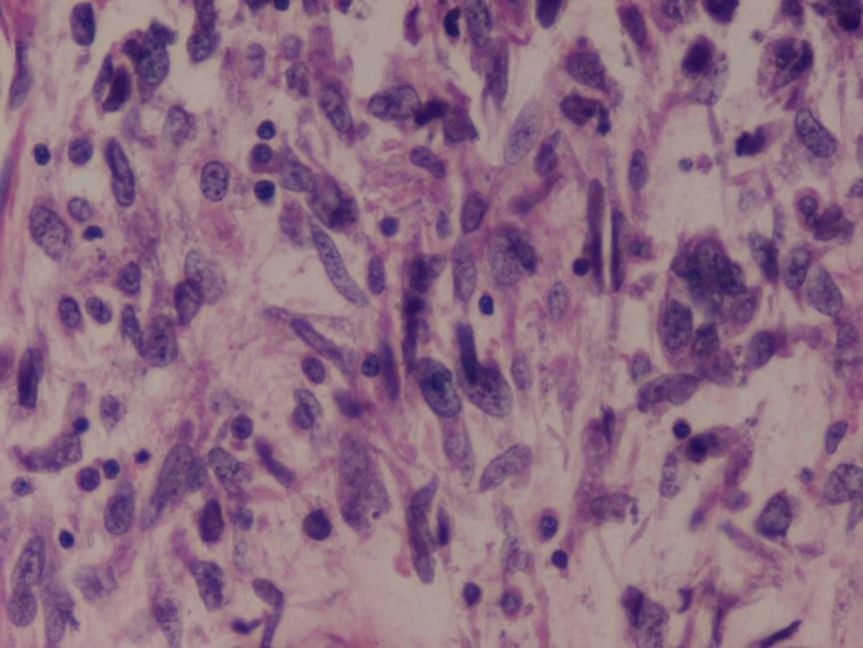
MTSCC: Foci of sarcomatoid differentiation were seen with pleomorphic high-grade nuclei without any evidence of vascular invasion. Haematoxylin and eosin, 40×.

**Figure 3.**
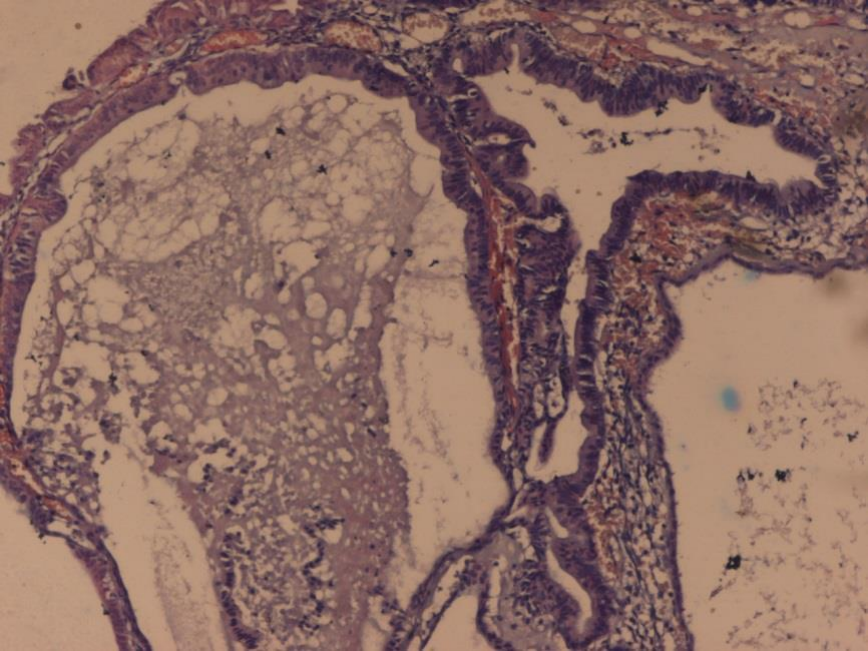
The typical blue-coloured mucin in the stroma between the tubules and the cords. Alcian blue stain, 40×.

**Figure 4.**
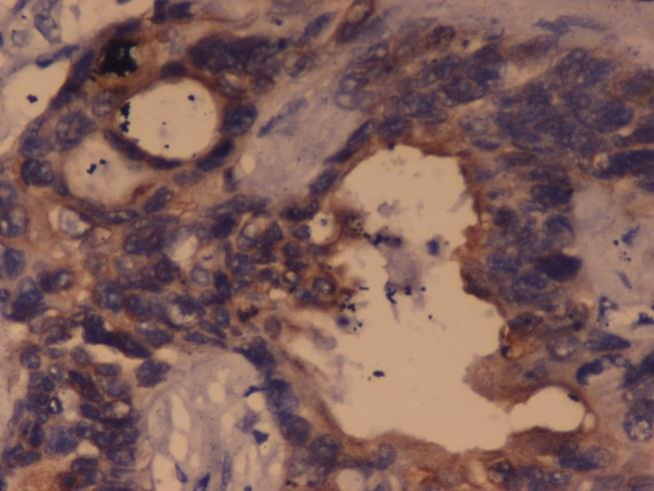
MTSCC: Immunohistochemistry showing diffuse positivity for cytokeratin in the tumour cells. IHC cytokeratin, 40×.

The operated specimen was encapsulated, solid and cystic with visible areas of capsular breach, of size 21 × 18 × 7 cm, and encased the upper pole and the middle region of the kidney. Perirenal fat and Gerota’s fascia were not involved. Microscopically, the tumour consisted of elongated, anastomosing tubules separated by lakes of mucin from a prominent spindle cell area. The tubules were composed of low cuboidal cells with amphophilic to eosinophilic cytoplasm with mild anisonucleosis. Foci of SD comprising about 30% of the tumour were seen, with pleomorphic low-grade nuclei. No lymphovascular invasion was noted (**Figures 1 and 2**). Alcian blue stain showed the typical blue-coloured mucin in the stroma between the tubules and the cords (**Figure 3**). The tumour cells were diffusely positive for cytokeratin 7 (**Figure 4**), EMA and vimentin but negative for CD10. Six cycles of adjuvant chemotherapy (vincristine, Adriamycin and cyclophosphamide [VAC] regimen), comprising cisplatin 50 mg/m^2^, vincristine 1.0 mg/m^2^, Adriamycin 40 mg/m^2^/day (Dako, Germany), cyclophosphamide 1000 mg/m^2^ and actinomycin-D 0.5 mg/m^2^ followed by 50 Gy/25 fractions of cobalt-60 teletherapy were given. The patient tolerated the therapy well without any appreciable adverse effects and unremarkable blood chemistry findings. Follow-up after 12 months demonstrated that the patient was doing well with no evidence of recurrence or metastasis.

## Discussion

MTSCC is a low-grade tumour. It has a preponderance in women and has a good prognosis (5, 6). The age of presentation is quite varied, from 30 to 80 years (5, 6). The complaint presented is usually pain in the abdomen, as was observed in our patient (6–8). Since the recognition of MTSCC as an individual entity by the WHO, several variants of the tumour have been described, such as mucin-poor MTSCC and MTSCC with neuroendocrine differentiation (9, 10).

SD has been recognized in all types of RCC since the Heidelberg classification of renal cell tumours was published in 1997 (11). The sarcomatoid component has fibrosarcoma-like appearance, which is reported in 14–65% of cases (12). Three to 24% tumours have no pattern of appearance, while hemangiopericytoma-like pattern and malignant fibrosarcoma-like appearance have also been documented (11–13). Unlike the spindle sarcomatoid cells, the inherent spindle cell elements of MTSCC have distinctively low-grade cytology, and they occasionally blend with tubular structures and variable mucinous stroma. Immunohistochemistry of MTSCC shows that the neoplastic cells of both the tubules and the spindle cells are strongly positive for PAX 2/8, cytokeratin 7 and 8, EMA, AMACR and E-cadherin, with variable expression of vimentin and high-molecular-weight cytokeratin (14, 15). The sarcomatoid cells are associated with significant necrosis, marked nuclear pleomorphism, high mitotic activity, higher proliferation fraction (MIB1) and loss of AMACR or cytokeratin 7 expression (16, 17).

Ours is an unusual case of MTSCC with SD in a 57-year-old man; only a few of such cases have been described yet. Features like interconnecting tubules of low-grade cuboidal cells in a mucinous matrix with areas of benign spindle cells were diagnostic of MTSCC in our case. A distinct focus of high-grade morphology consistent with SD was seen. Diagnosis of MTSCC with SD was assisted by immunohistological findings, which showed strong positivity for AMACR, EMA, cytokeratin 7 and vimentin.

The differentials of MTSCC include papillary RCC, collecting duct carcinoma and metanephric adenoma. Papillary RCC lacks a spindle cell component and rarely shows lakes of mucin. Collecting duct carcinoma shows high-grade histological features and lacks mucin. Metanephric adenoma shows tubulopapillary architecture, but stromal and spindle cell components are lacking (12–15).

MTSCC with sarcomatoid differentiation has a poor prognosis despite the low malignant potential of MTSCC, with widespread metastasis to lymph nodes, bones and lungs in approximately 55.4% of the cases (5, 18). Distinction of the sarcomatoid histology from the inherent spindle cell component of MTSCC is important because of its unfavourable prognostic implication (18, 19). Dhillon et al (5) and Bulimbasic et al (20) have reported 5 cases of the sarcomatoid variant of MTSCCs with aggressive behaviour, of which distant metastases with fatal outcome were seen in 3 cases (20). This may suggest that sarcomatoid changes are related to the biobehaviour of MTSCCs (20). Therefore, the presence of SD is a harbinger of poor prognosis and must be reported in any type of RCC. The presence of spindle cells in MTSCC may be confused with small areas of SD, so adequate sampling and careful histological examination are required in all MTSCC cases (8). It is essential that areas of atypical spindle cells, especially when associated with necrosis, should be reported and the possibility of SD considered (8, 15). Thus, it is essential to search for areas of sarcomatoid differentiation in the case of MTSCC so that complete surgical resection in the form of radical nephrectomy and radiation therapy is employed, followed by regular clinical and radiological follow-up to exclude possible metastatic disease (19, 20).
